# A translation-independent function of PheRS activates growth and proliferation in *Drosophila*

**DOI:** 10.1242/dmm.048132

**Published:** 2021-03-18

**Authors:** Manh Tin Ho, Jiongming Lu, Dominique Brunßen, Beat Suter

**Affiliations:** Institute of Cell Biology, University of Bern, Baltzerstrasse 4, Bern 3012, Switzerland

**Keywords:** Aminoacyl tRNA synthetase, PheRS, FARSA, Growth and proliferation control

## Abstract

Aminoacyl transfer RNA (tRNA) synthetases (aaRSs) not only load the appropriate amino acid onto their cognate tRNAs, but many of them also perform additional functions that are not necessarily related to their canonical activities. Phenylalanyl tRNA synthetase (PheRS/FARS) levels are elevated in multiple cancers compared to their normal cell counterparts. Our results show that downregulation of PheRS, or only its α-PheRS subunit, reduces organ size, whereas elevated expression of the α-PheRS subunit stimulates cell growth and proliferation. In the wing disc system, this can lead to a 67% increase in cells that stain for a mitotic marker. Clonal analysis of twin spots in the follicle cells of the ovary revealed that elevated expression of the α-PheRS subunit causes cells to grow and proliferate ∼25% faster than their normal twin cells. This faster growth and proliferation did not affect the size distribution of the proliferating cells. Importantly, this stimulation proliferation turned out to be independent of the β-PheRS subunit and the aminoacylation activity, and it did not visibly stimulate translation.

This article has an associated First Person interview with the joint first authors of the paper.

## Abstract (precis)

**Summary:** A moonlighting activity of the α-subunit of the Phenylalanyl tRNA synthetase in *Drosophila* promotes growth and proliferation through a novel mechanism that neither involves aminoacylation nor translation.

## INTRODUCTION

Many cancer tissues display higher levels of phenylalanyl transfer RNA (tRNA) synthetase (PheRS; also known as FARS) than their healthy counterparts according to the database ‘Gene Expression across Normal and Tumor tissues 2’ (GENT2) (Park et al., 2019). Interestingly, a correlation between tumorigenic events and PheRS expression levels had been noted much earlier for the development of myeloid leukemia (Sen et al., 1997). Despite this, a possible causative connection between elevated PheRS levels and tumor formation had so far not been reported and, to our knowledge, also not been studied. This might have been due to the assumption that higher PheRS levels could simply reflect the demand of tumorigenic cells for higher levels of translation, or it could have to do with the difficulty of studying the moonlighting function of a protein that is essential in every cell for basic cellular functions such as translation.

Aminoacyl tRNA synthetases (aaRSs) are important enzymes that act by charging tRNAs with their cognate amino acid, a key process for protein translation. This activity makes them essential for accurately translating the genetic information into a polypeptide chain (Schimmel and Söll, 1979). Besides their well-known role in translation, an increasing number of aaRSs have been found to perform additional functions in the cytoplasm, the nucleus and even outside the cell (Guo and Schimmel, 2013; Nathanson and Deutscher, 2000; Smirnova et al., 2012; Casas-Tintó et al., 2015; Gomard-Mennesson et al., 2007; Greenberg et al., 2008; Otani et al., 2002; Zhou et al., 2014). Moonlighting aaRSs regulate alternative splicing, RNA processing and angiogenesis (Lee et al., 2004). For example, the amino acid-binding site of LysRS has an immune response activity, and TrpRS inhibits vascular endothelial (VE)-cadherin and through this elicits anti-angiogenesis activity (Tzima et al., 2005; Yannay-Cohen et al., 2009).

Cytoplasmic PheRS is one of the most complex members of the aaRSs family, a heterotetrameric protein consisting of two alpha (α) and two beta (β) subunits responsible for charging tRNA^Phe^ during translation (Roy and Ibba, 2006). The α-subunit includes the catalytic core of the tRNA synthetase, and the β-subunit has structural modules with a wide range of functions, including tRNA anti-codon binding, hydrolyzing mis-activated amino acids and editing misaminoacylated tRNA^Phe^ species (Ling et al., 2007; Lu et al., 2014; Roy and Ibba, 2006). Importantly, both subunits are needed for aminoacylation of tRNA^Phe^.

We set out to address the question whether and how elevated levels of PheRS can contribute to tumor formation. To test for this activity, we studied the role of PheRS levels in the *Drosophila* model system, with the goal of finding out whether elevated levels of PheRS allow higher translation activity or whether a moonlighting role of *PheRS* might provide an activity that contributes to elevated growth and proliferation*.* We found that α-PheRS levels regulate cell proliferation in different tissues and cell types. Interestingly, however, elevated levels of α-PheRS do not simply allow higher levels of translation. Instead, α-PheRS performs a moonlighting function by promoting proliferation independent of the β-PheRS subunit, even if it lacks the aminoacylation activity.

## RESULTS

### PheRS is needed for proliferation and for normal organ and animal growth

**Fig. 1.**
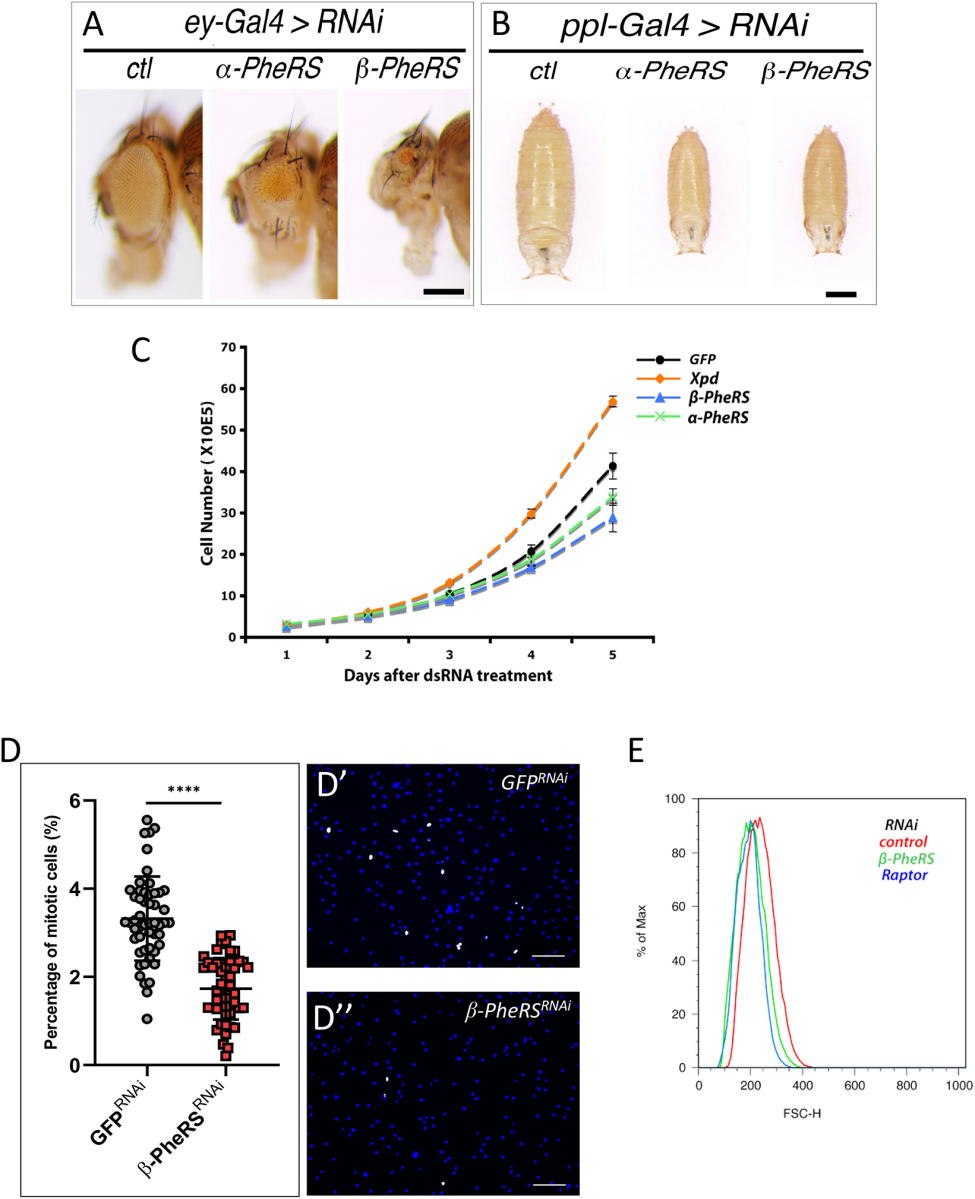
**PheRS knockdown reduces cell proliferation and tissue size.** (A,B) RNAi knockdown of *PheRS* subunits in fly eyes (A) and fat bodies (B). *ey-Gal4* (*ey-Gal4/+; UAS-PheRS^RNAi^/+*; A) and *ppl-Gal4* (*ppl-Gal4/+; UAS-PheRS^RNAi^/+*; B) were used to drive RNAi expression. The controls were GFP RNAi. RNAi knockdown of either subunit reduced the eye size (A; scale bar: 250 µm) and the size of the entire pupae (B; scale bar: 500 µm). (C-E) Proliferation, cell cycle distribution and cell size analysis of Kc cells upon knockdown of PheRS. Knockdown of either subunit reduces PheRS in Kc cells (Lu et al., 2014). Here, *β-PheRS* knockdown was used. RNAi knockdown was carried out by directly adding dsRNA to the medium. (C) For analysis of proliferation, *Xpd* RNAi was used as a control. Cells were harvested on days 1, 2, 3, 4 and 5 after dsRNA treatment. (D-D″) RNAi knockdown reduces the mitotic index. The mitotic index was determined by counting the phospho-Histone H3-positive cells (white dots in D′,D″) and all cells. Over 10,000 cells were counted for each treatment. ****P<0.0001 (unpaired Student's *t*-test). Scale bars: 25 μm. (E) Reduced PheRS decreases the cell size of Kc cells. Cell size was determined by measuring the forward scatter (FSC) in the FACS analysis. *raptor* RNAi was the positive control, and *β-PheRS* knockdown showed a similar cell size distribution.

The *Drosophila* FARS homolog PheRS is a hetero-tetrameric aaRS consisting of two α- and two β-subunits encoded by *α-PheRS* and *β-PheRS*, which are essential genes in *Drosophila* (Lu et al., 2014). To find out whether cellular levels of α-PheRS correlate with and possibly contribute to growth, we tested whether reduced levels in specific tissues affect growth of the organ and animal. For this we used RNA interference (RNAi) to reduce their activity in two specific tissues: the eye, an organ that is not essential for viability, and the fat body (Fig. 1A,B). Indeed, knocking down either of the two subunits in the developing eye reduced the size of the adult eye (Fig. 1A). Similarly, reducing *α-PheRS* or *β-PheRS* expression levels in the larval fat body caused a growth reduction. However, presumably because of its role in systemic growth (Texada et al., 2020), the fat body knockdown of *PheRS* reduced the size of the entire pupae (Fig. 1B).

To further analyze the changes at the cellular level, the effect of knocking down *α-PheRS* and *β-PheRS* in *Drosophila* Kc cells was first examined at the level of cell proliferation (Fig. 1C). The knockdowns were carried out by adding double-stranded RNA (dsRNA) into the medium, and the cell numbers were recorded over the following days. Compared to the controls, cells treated with *β-PheRS* RNAi started to show lower cell numbers on day 3, and the cell count was ∼75% of that of the control on day 5. In Kc cells, knocking down either subunit alone reduced levels of the α-PheRS and β-PheRS subunit (Lu et al., 2014). It was therefore reassuring that *α-PheRS* knockdown showed similar results. Because routine cell viability assays did not point to an increase in dead cells in any of the samples upon RNAi treatment, a change in cell numbers should reflect a proliferation change. As a positive control, *Xpd* RNAi was performed and showed the published increase in cell proliferation (Chen et al., 2003). The fact that knockdown of *Xpd* can speed up cell growth and proliferation not only indicates that the Kc cells were healthy, but also that the PheRS levels are not limiting for growth and proliferation, but can sustain even higher proliferative activity.

Determining the mitotic index upon *PheRS* knockdown revealed that the reduced PheRS levels caused a strong reduction in mitotic cells as indicated by the lower fraction of phospho-Histone 3 (PH3; mitotic marker)-positive cells. The RNAi treatment reduced the mitotic index to 1.7±0.16%, which corresponded to half of the control (3.3±0.16%) (Fig. 1D). Similarly, cell size was also affected by *PheRS* knockdown (Fig. 1E). The cell size showed a reduction that was similar to the one observed upon knocking down *raptor* with RNAi. Raptor is a component of the TORC1 (target of rapamycin complex 1) signaling that regulates cell growth (Kim et al., 2002). These experiments showed that *α-PheRS* and *β-PheRS* are needed for normal growth and proliferation of cells, organs and entire animals. This result might reflect the requirement for the enzymatic activity of PheRS in charging the tRNA^Phe^ with its cognate amino acid phenylalanine, or it might point to a novel, possibly moonlighting function of PheRS in stimulating growth and proliferation.

### PheRS lacks apparent amino acid sensor activity for TORC1

**Fig. 2.**
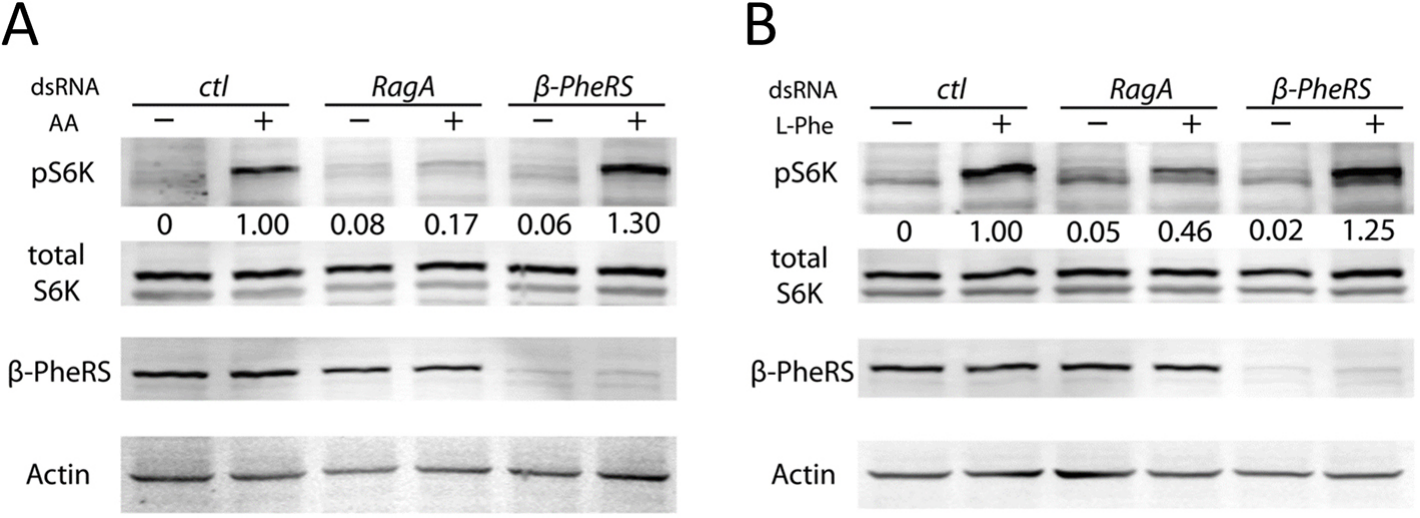
**PheRS does not act as an amino acid sensor for the TORC1 complex.** (A) *β-PheRS* knockdown cannot block the TORC1 complex from sensing the availability of amino acids. Phospho-S6K was used as a readout of TORC1 signaling. Starvation (−) was performed by depriving cells of amino acids (AA) for 30 min, and stimulation (+) was performed by re-adding amino acids for 30 min after starvation. The control was a mock RNAi, *RagA* RNAi is known to block the sensing of amino acids. Phospho-S6K levels were quantified relative to the Actin levels in the same extract. (B) *β-PheRS* knockdown did not block the TORC1 complex from sensing the availability of L-Phe. The same experiment as in A was performed, but using L-Phe and L-Glu for stimulation. L-Glu was reported to be necessary for amino acid transport (Nicklin et al., 2009). Levels of phospho-S6K were quantified relative to the Actin levels in the same extract.

The TORC1 signaling pathway activates growth and proliferation of cells depending on the availability of amino acids, growth factors and energy (Laplante and Sabatini, 2012; Wullschleger et al., 2006). In addition to its canonical function in charging tRNA^Leu^ with leucine, the LeuRS serves as the central amino acid sensor in this pathway (Bonfils et al., 2012; Han et al., 2012). We therefore tested whether PheRS might also be involved in nutrient sensing for TORC1 signaling in an analogous way. Amino acid deprivation causes a downregulation of phosphorylation of dS6K in Kc cells, and subsequent stimulation with amino acids restores phospho-dS6K levels (Kim et al., 2008). The Rag complex was identified as a nutrient sensor in this pathway, and knockdown of *RagA* (also known as *RagA-B*) prevents the TORC1 complex from sensing the availability of amino acids (Kim et al., 2008; Sancak et al., 2008). We therefore used *RagA* as our control (Fig. 2A,B). In contrast to *RagA*, knocking down *β-PheRS* did not prevent amino acid sensing in this assay, and phosphorylation of dS6K was still induced to a similar level as in the control when amino acids were re-added after deprivation (Fig. 2A,B). In this case, it did not matter whether we re-added all amino acids or only L-Phe. Although we cannot rule out that the RNAi knockdown was insufficient to reveal an amino acid-sensing function for PheRS, the results seem to suggest that PheRS might not serve as an amino acid sensor upstream of the TORC1 complex.

### Non-canonical α-PheRS activity is sufficient to induce additional M-phase cells

**Fig. 3.**
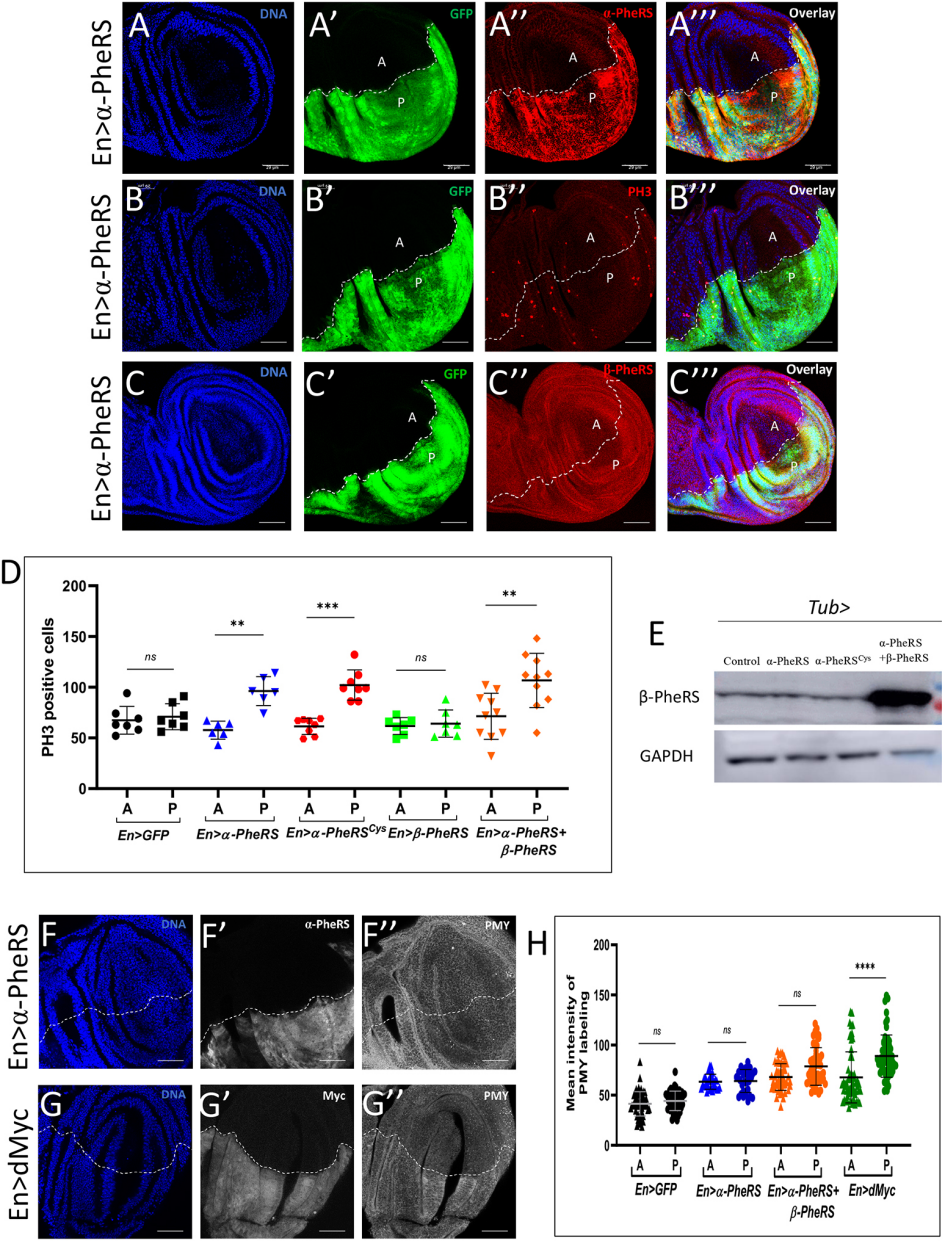
**Elevated α-PheRS promotes** **the** **appearance of mitotic cells without *β-PheRS* and without stimulating translation.** (A-C‴) Wing disc phenotypes induced by the overexpression of *α*-*PheRS* or *α*-*PheRS^Cys^*. *en-Gal4* was used to drive transgene expression in the posterior compartment of developing wing discs [*en-Gal4/+;UAS-GFP/UAS-α-PheRS^(Cys)^*]. Mitotic cells were visualized with anti-phospho-Histone H3 (PH3) antibodies. A, anterior compartment; P, posterior compartment. Scale bars: 29 μm. (D) Mitotic (PH3-positive cells) cells in the posterior (P) compartment increased 40% relative to the anterior (A) one when both α-PheRS and β-PheRS or only the α-PheRS subunit alone were raised. *n*=10, **P<0.01, ***P<0.001; ns, not significant (paired Student's *t*-test). (E) Whereas increasing the expression of α-PheRS and β-PheRS together showed increased β-PheRS levels, elevating the α-PheRS subunit alone did not affect the β-PheRS subunit levels. (F-G″) Protein synthesis did not increase upon overexpression of *α-PheRS* or *α-PheRS* and *β-PheRS* together*. en-Gal4* was used to drive the overexpression of the transgenes in the posterior compartment of wing discs. dMyc was used as a positive control (*en-Gal4/UAS-Myc::MYC;UAS-GFP/+*). Scale bars: 29 μm. (H) Protein synthesis was measured by the mean intensity of the puromycin (PMY) signal labeling the nascent polypeptides. *n*=15, ****P<0.0001; ns, not significant (paired Student's *t*-test).

**Fig. 4.**
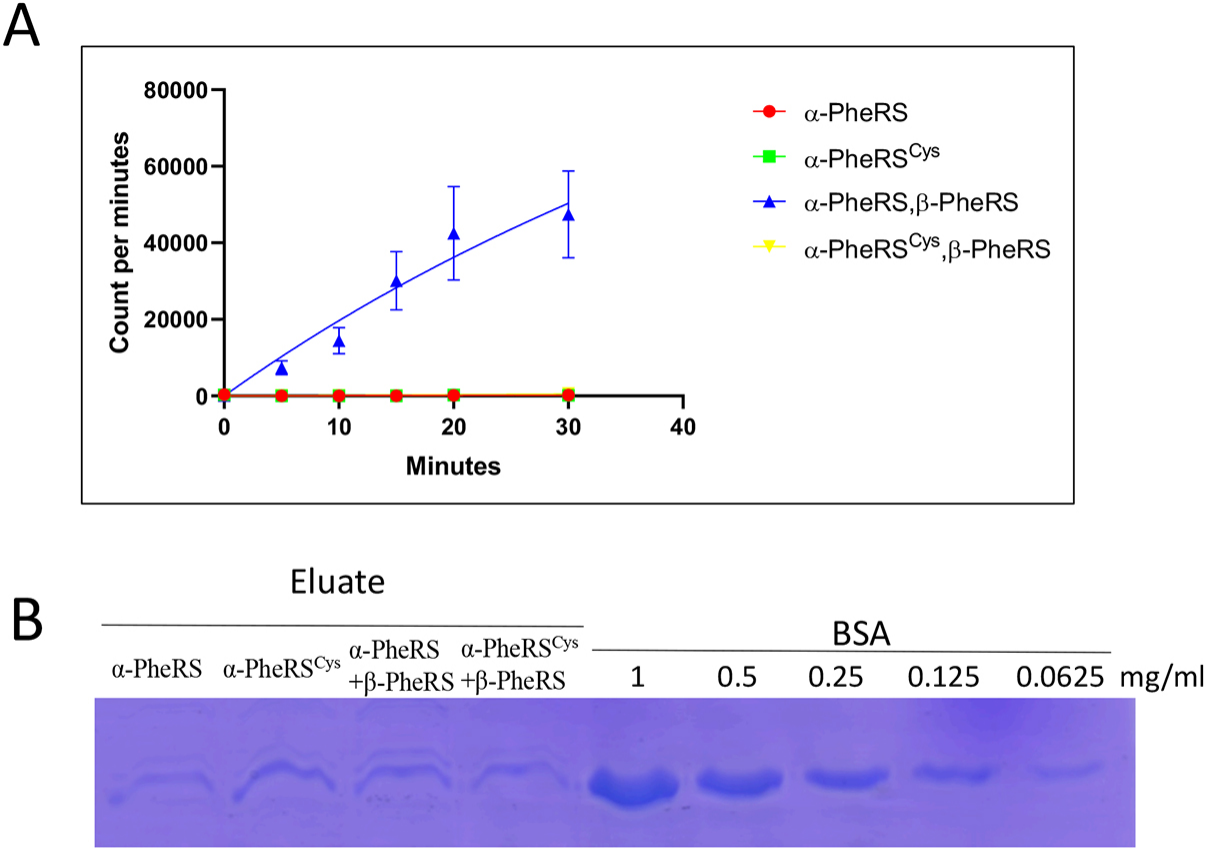
**The *α-PheRS^Cys^* mutant does not support aminoacylation *in vitro*.** The aminoacylation assay was performed with the mixture of the recombinant protein (α-PheRS or α-PheRS^Cys^ and β-PheRS) simultaneously expressed in *E. coli*. tRNA^Phe^ from yeast was aminoacylated with [^3^H] phenylalanine. (A) The [^3^H] phenylalanine decays were counted in a scintillation counter. In this assay, wild-type α-PheRS+β-PheRS subunits together gave a rise in counts per minute (CPM) of up to 47,417, and α-PheRS^Cys^+β-PheRS produced less than 654 CPM. α-PheRS or α-PheRS^Cys^ alone produced 210 and 191 CPM, respectively. (B) Purified recombinant protein and protein complexes were controlled and quantified on Coomassie Blue-stained gels using bovine serum albumin (BSA) as a standard. The clear bands indicate that the Cys mutation did not affect the solubility of the recombinant proteins.

Circumstantial evidence suggests that elevated PheRS levels do not simply allow higher translational activity to overcome a growth rate restriction imposed by hypothetically limiting levels of PheRS. PheRS is unlikely to be rate limiting for cellular growth because animals with only one copy of *α-PheRS* (*α-PheRS*/–) or *β-PheRS* (*β-PheRS*/–) do not show a phenotype (Lu et al., 2014). Furthermore, Kc tissue culture cells can be stimulated to grow more rapidly without stimulating the expression of *α-PheRS* or *β-PheRS* (Fig. 1C; Chen et al., 2003). To test whether elevated levels of PheRS can stimulate growth or proliferation, we expressed *α-PheRS*, *β-PheRS* and both subunits together in the posterior compartment of wing discs using a Gal4 driver under the control of the *engrailed* (*en*) promoter. This *en-Gal4* drives the expression of the *α-PheRS* and *β-PheRS* open reading frames (ORFs) that were cloned behind a UAS promoter and integrated into a defined landing platform in the fly genome that also contains the *α-PheRS* and *β-PheRS* genes. In this assay, the anterior compartment expresses normal endogenous PheRS levels and serves as an internal control, and the expression of, for instance, α-PheRS driven by *en-Gal4* increased the level of α-PheRS signal by 80% (Fig. S1). Depending on the specificity of the antibody, the actual increase in α-PheRS levels might be somewhat higher (for details, see Fig. S1). When α-PheRS and β-PheRS levels were raised in the posterior wing disc compartment together, the PH3 labeling revealed a 40% increase in mitotic cells in the posterior compartment relative to the anterior one of the same wing disc (Fig. 3). Surprisingly, the same result was obtained when only the levels of the α-PheRS subunit were raised (Fig. 3A-D), but not when only the β-PheRS subunit levels were raised (Fig. 3D). In addition, raising the α-PheRS subunit alone did not affect the β-PheRS subunit levels (Fig. 3C-C‴,E). Furthermore, aminoacylation requires both PheRS subunits to ligate Phe to the tRNA^Phe^ (Fig. 4A). The fact that elevated α-PheRS levels alone are sufficient to raise the mitotic index is therefore another strong indication that α-PheRS possesses a proliferative activity that is unlikely to be mediated by increased tRNA^Phe^ aminoacylation.

To test directly whether α-PheRS can increase proliferation without stimulating translation, we made a mutant version of α-PheRS in which Tyr412 and Phe438 are replaced by cysteine (Cys). These substitutions are predicted to block the entrance into the phenylalanine binding pocket, preventing binding of Phe and aminoacylation of tRNA^Phe^ by the mutant PheRS^Cys^ (Finarov et al., 2010). To test whether the PheRS^Cys^ substitution indeed reduces the aminoacylation activity of PheRS, we expressed mutant and wild-type α-PheRS subunits individually or together with β-PheRS subunits in *Escherichia coli*, His tag purified them and performed aminoacylation assays with the appropriate amount of recombinant soluble proteins that were normalize to bovine serum albumin (BSA) standards (Fig. 4B). The clear band in Fig. 4B showed that the Cys mutation did not affect the solubility of the recombinant proteins. α*-*PheRS^Cys^ together with wild-type β-PheRS produced the same background signal as the α-PheRS subunit alone, and this signal was clearly lower than the one obtained with wild-type α-PheRS plus β-PheRS (Fig. 4A). The *Drosophila* gene encoding the cytoplasmic α-PheRS subunit is located on the X chromosome. A P-element insertion into the 5′-untranslated region of the *α-PheRS* transcript (*α-PheRS^G2060^*) causes recessive lethality that can be rescued by a genomic copy of wild-type *α-PheRS* (*gα-PheRS*) that supports aminoacylation (Lu et al., 2014). A genomic copy of the aminoacylation-defective α-*PheRS^Cys^*, *gα-PheRS^Cys^*, did not rescue the lethality of the *α-PheRS^G2060^* mutant, indicating that the Cys mutant is indeed not functional in aminoacylation *in vivo* in *Drosophila*. Despite this apparent lack of aminoacylation activity, expressing a transgenic copy of *α*-*PheRS^Cys^* in the posterior compartment of the wing disc with the *en-Gal4* driver caused a 67% increase in the number of mitotic cells in the above assay (Fig. 3D). The fact that the mutant *α*-*PheRS^Cys^* version caused an increase in mitotic cells at least as strongly as the wild-type *α*-*PheRS* expressed in the same way, together with the fact that β-PheRS overexpression was not needed for this effect, strongly suggests that the increased mitotic index is promoted without increasing the canonical function of PheRS.

We also tested directly whether elevated expression of wild-type *α*-*PheRS* and expression of *α-PheRS* and *β-PheRS* together are indeed unable to cause elevated translation as we expected. For this, we analyzed protein synthesis activity in the two wing compartments by puromycin (PMY) staining using the ribopuromycylation method (RPM) (Deliu et al., 2017). Testing this method, we first expressed elevated levels of the transcription factor dMyc (also known as Myc) in the posterior wing disc compartment. This positive control led to increased protein synthesis activity and anti-PMY signal in the dMyc-overexpressing posterior compartment relative to the anterior compartment of the same discs. In contrast, neither *en-Gal4*-driven expression of α-PheRS alone nor combined expression with β-PheRS increased the puromycin labeling in the posterior compartment (Fig. 3F-H). The combined results therefore demonstrate unambiguously that elevated α-PheRS levels cause additional cells to be in mitosis through a non-canonical mechanism that does not involve a general increase in translation.

We also considered the remote possibility that the elevated expression of only one PheRS subunit might alter the ratio of Phe-tRNA^Phe^/tRNA^Phe^ (charged tRNA^Phe^ to uncharged tRNA^Phe^) and that this might affect the translatability of specific mRNAs with a very high or a very low frequency of Phe codons. We therefore used a proteomics approach to study the effect of overexpressing *α-PheRS* with the strong ubiquitous *tub-Gal4* driver. We then analyzed the expression of the *Drosophila* proteome and correlated it to the Phe frequency in these proteins (Fig. S2A,B). The results showed that there was no, or only a minor, negative correlation (−0.0029 using the linear and −0.030 with the log2 expression data). In conclusion, we could not find evidence that the unbalanced expression of only one PheRS subunit markedly changes the efficiency of reading Phe codons.

The non-canonical activity emanating from α-PheRS or α-PheRS^Cys^ is capable of inducing more cells to be in mitosis. Such a phenotype is likely to come about by specifically slowing down progression through M-phase, causing higher numbers of cells to remain in the PH3-positive state. Alternatively, the activity might either promote proliferation of mitotic cells, or induce proliferation in non-cycling cells.

### α-PheRS levels accelerate growth and proliferation

**Fig. 5.**
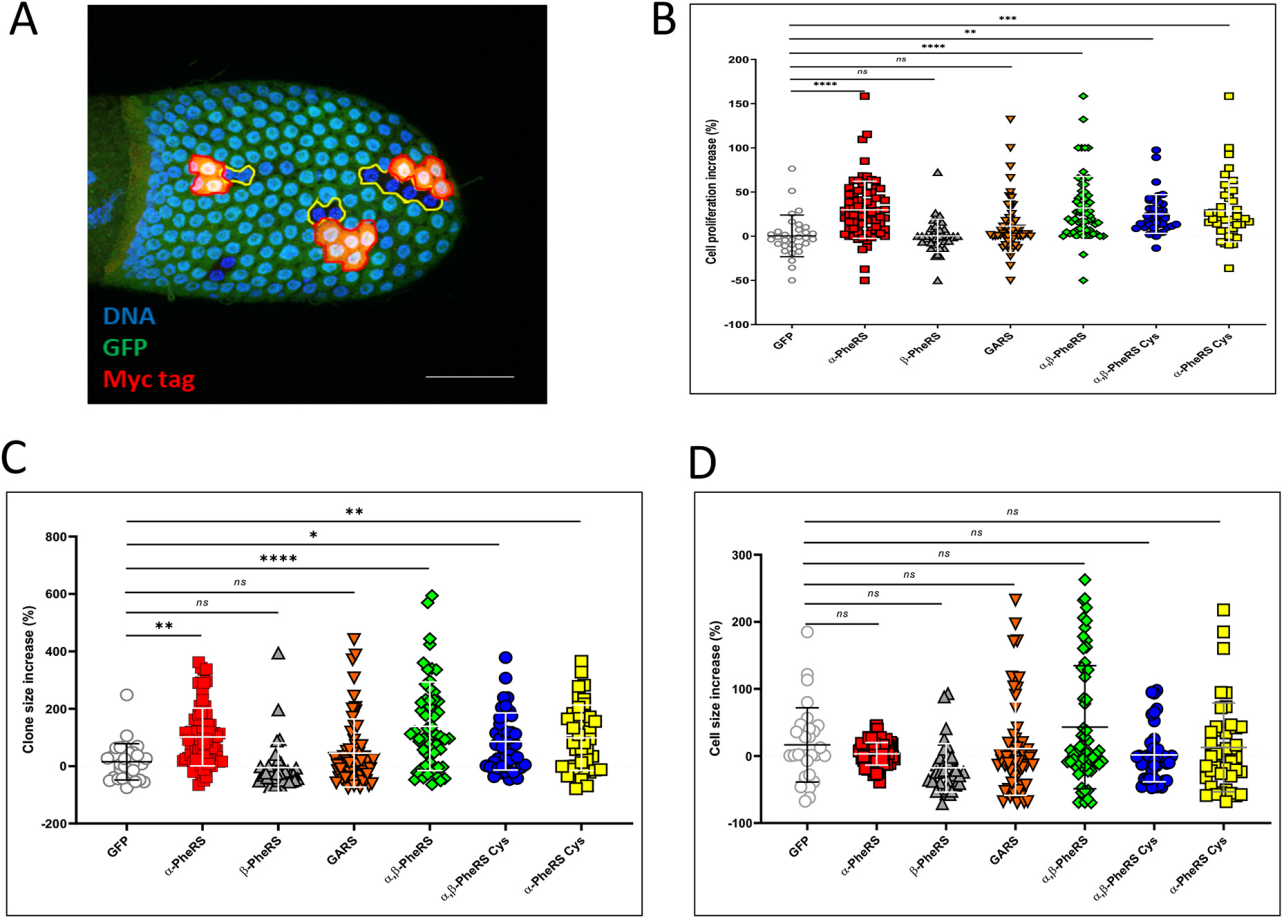
***α-PheRS* overexpression promotes** **follicle cell proliferation independent of tRNA^Phe^ aminoacylation.** Clonal analysis (twin spot) experiment of the effect of overexpression of the PheRS subunits in follicle cells by the MARCM technique [*hspFLP/+;Act-Gal4/+;neoFRT82B,tub-Gal80/neoFRT82B,UAS-α-PheRS^(Cys)^*]. After inducing mitotic recombination by expressing the FLPase under the control of the heat-shock (*hs*) promoter at 37°C for 40 min, a recombining cell will divide and give rise to two proliferating clones. (A) One clone (red) overexpresses PheRS; its twin clone (GFP^−^ signal) does not and expresses normal levels (internal control, outlined with yellow). Scale bar: 29 μm. (B) Three days after inducing the recombination, the average number of cell divisions was calculated for each clone and compared to its twin spot to obtain the proliferation increase (%). (C) Three days after inducing the recombination, the average clone size was calculated for each clone and compared to its twin spot to obtain the clone size increase (%). (D) Three days after inducing the recombination, the average size of a single cell in each clone was calculated and compared to its twin spot to obtain the cell size increase (%). *n*=30, *P<0.05, **P<0.01, ***P<0.001, ****P<0.0001; ns, not significant (one-way ANOVA).

To test the effects of PheRS levels on growth and proliferation directly and in an additional cell type, we set up ‘mosaic analysis with repressible cell marker’ (MARCM; Wu and Luo, 2006) assays in the ovarian follicle cells. Twin spot clones were generated with one clone expressing elevated levels of PheRS and the GFP marker, and its twin clone expressing normal endogenous levels of PheRS and serving as an internal control (Fig. 5A). The results of this experiment showed that clonally elevated levels of both subunits of PheRS accelerated cell proliferation, on average, by 32% (Fig. 5B). In contrast, clonal elevated expression of GFP with only the β-PheRS subunit or with GlyRS (also known as GARS) did not significantly promote clonal expansion (Fig. 5B). This confirms that the stimulation of proliferation is specific for PheRS and not a general role of aaRSs. Interestingly, clonal elevated expression of GFP with the α-PheRS subunit alone also stimulated cell proliferation autonomously by 30% (Fig. 5B), and, intriguingly, this was very close to the 32% increase calculated for the clone overexpressing both PheRS subunits (Fig. 5B). Remarkably, the increase in number of mitotic cells observed upon α-PheRS overexpression in the posterior compartment of the larval wing discs (Fig. 3C) was in a comparable range to the proliferation increase in the follicle cell assay (Fig. 5B). These results therefore suggest that α-PheRS levels promote cell proliferation and that α-PheRS levels have this activity in different tissues. Interestingly, the *tub-Gal4* driver used in this experiment to produce the elevated α-PheRS levels was found to cause only a 57% increase in α-PheRS levels in L1 larvae when used to solely express this subunit (Fig. S2C).

Similarly, clonal elevated expression of *α-PheRS^Cys^* alone and *α-PheRS^Cys^* together with *β-PheRS* (*PheRS^Cys^*) stimulated cell proliferation in the follicle cell twin spot experiment by 28% and 25%, respectively (Fig. 2B), again confirming that this effect does not depend on increased canonical PheRS activity.

We also measured the clone size of twin spot clones to find out whether the elevated PheRS could enhance cell growth. This was accompanied by results on cell proliferation that clonally elevated levels of both subunits of PheRS or single α-PheRS subunit augmented clone size by 139% and 102%, respectively, whereas elevated levels of single β-PheRS subunit or GlyRS did not result in significant increase in clone size (Fig. 5C). We also observed that the size of single cells in twin spot clones remained unchanged (Fig. 5D), whereas the total clone sizes increased according to the increase in cell number per clone (Fig. 5B,C), indicating that cell size control was not affected. These results therefore suggest that α-PheRS levels promote cell growth and proliferation and that α-PheRS levels have this activity in different tissues.

Alternatively, elevated *α-PheRS^Cys^* alone and *α-PheRS^Cys^* together with *β-PheRS* (*PheRS^Cys^*) stimulated cell proliferation in the follicle cell twin spot experiment by 28% and 25%, respectively (Fig. 5B,C). The clonal sizes also expanded by 99% and 86%, respectively, by accelerating levels of α-PheRS^Cys^ alone and α-PheRS^Cys^ together with β-PheRS. These observations again confirmed that this effect on cell growth and proliferation does not depend on increased canonical PheRS activity.

## DISCUSSION

Our work revealed that PheRS not only charges tRNAs with their cognate amino acid Phe, but that it also performs a moonlighting function in stimulating cellular growth and proliferation. Because levels of α-PheRS are elevated in many tumor cells compared to their healthy counterparts, and because a positive correlation between these levels and tumorigenic events had been noted some time ago (Sen et al., 1997), it was important to find out whether elevated PheRS levels are a mere consequence of the high metabolic activity of the tumor cells or whether they might also contribute to overproliferation of tumor cells. Here, we showed that α-PheRS has the potential to promote growth and proliferation and that it can do this independent of its aminoacylation activity, i.e. through a non-canonical or moonlighting activity. We found unambiguous evidence for the non-canonical nature of this proliferative activity and also showed that general translation is not elevated when this phenotype is induced by elevated α-PheRS or even the entire PheRS protein (Fig. 3D-D″). Aminoacylation of tRNA^Phe^ requires both subunits to form the tetrameric protein α_2_β_2_-PheRS that can aminoacylate tRNA^Phe^ (Fig. 4), and overexpression of an aminoacylation-dead *α-PheRS^Cys^* mutant subunit alone (without simultaneous overexpression of the β-PheRS subunit) increased the cell numbers in the follicle cell clones as much as the wild-type gene expressed in the same way did (Fig. 4 and Fig. 5B). The described non-canonical functions of α-PheRS are not only independent of the aminoacylation activity, they also do not reflect a function in sensing the availability of its enzymatic substrate, Phe, for the major growth controller, the TOR kinase, like the aaRS members TrpRS or LeuRS (Fig. 2) (Adam et al., 2018; Bonfils et al., 2012; Han et al., 2012).

The notion that the α-PheRS subunit can be stable (Fig. 3A″) and function independently of the β-subunit (Fig. 3C-C‴,E) was surprising because previous results showed that the two subunits were dependent on the presence of the other subunit for their stability (Antonellis et al., 2018; Lu et al., 2014; Xu et al., 2018). Our results now show that this requirement does not apply to all cell types. In mitotically active follicle cells, the posterior compartment of the wing disc and possibly other cells, elevated expression of the α-PheRS subunit alone results in higher levels of α-PheRS accumulation and this produced a strong phenotype. This suggests that the α-PheRS and β-PheRS subunits function together in every cell to aminoacylate tRNA^Phe^, but, in addition, the α-subunit can be stable in specific cell types that appear to have retained their mitotic potential. In these cells, α-PheRS assumes a novel function in promoting cell growth and proliferation. This would then suggest that many differentiating cell types start to put a system in place that prevents α-PheRS accumulation at high levels. Such a mechanism could then contribute to reducing the proliferative activity of differentiated cells.

If α-PheRS does not act as an amino acid sensor for TORC1, how could it perform this function in growth and proliferation? The report that aaRSs can modify other proteins on Lys side chains with the amino acid they usually attach to the tRNA caused us initially to investigate this possibility (He et al., 2018). However, our results that the enzymatic activity of PheRS is not required for this effect strongly argued against such a pathway. Trying to find possible downstream targets of α-PheRS, we went through the list of proteins for which levels significantly increased or decreased in response to the elevated expression of this subunit (Table S1). For only one of these proteins, Psi, a role in growth and proliferation had been described (Guo et al., 2016). However, this protein becomes significantly underexpressed by elevated α-PheRS levels and this would be predicted to cause reduced growth and proliferation. This, like many other changes in the expression pattern, is therefore more easily explained by a more indirect effect, like the consequence of increased growth and proliferation (Table S1). Interestingly, the 3D structure of monomeric α-PheRS in the Swiss-Model online tool (Biozentrum, University of Basel; https://swissmodel.expasy.org/repository/uniprot/Q9W3J5) revealed that the α-PheRS consists of two distinct parts (Finarov et al., 2009): the catalytic core with aminoacylation function and, separated by a linker, another part with DNA-binding domains (DBDs) and unknown function (Finarov et al., 2010). Furthermore, the DBDs contain a predicted nuclear localization sequence (NLS) at position 159 (ADFKKRKLLQE) (http://nls-mapper.iab.keio.ac.jp/cgi-bin/NLS_Mapper_form.cgi). This region could be a candidate for the one that performs the non-canonical function of α-PheRS, and its structure might suggest that its activity involves entering the nucleus and binding to DNA, or possibly RNA, to stimulate growth and proliferation.

PheRS is not the only aaRS family member with roles beyond charging tRNAs (Dolde et al., 2014; Guo et al., 2010; Lu et al., 2015; Pang et al., 2014). For instance, MetRS/MRS is capable of stimulating ribosomal RNA (rRNA) synthesis (Ko et al., 2000), GlnRS/QRS can block the kinase activity of apoptosis signal-regulating kinase 1 (ASK1) (Ko et al., 2001), and a proteolytically processed form of YARS/TyrRS acts as a cytokine (Casas-Tintó et al., 2015; Greenberg et al., 2008). aaRSs are, however, not the only protein family that evolved to carry out more than one function. In recent years, it has become increasingly evident that many, if not most, proteins have evolved to carry out not only one, but two or more functions, providing interesting challenges in determining which of their activities are important for the specific function of a gene (Dolde et al., 2014).

Improper expression of PheRS was suspected long ago to promote carcinogenesis, but until now the mechanisms behind this effect remained unknown (Sen et al., 1997). Elevated mRNA levels of the human *α-PheRS*, *FARSA*, during the development of myeloid leukemia correlate with tumorigenic events. The GENT2 database published in 2019 also describes strong positive correlations between PheRS subunit mRNA levels and tumorigenic events in several tissues and cancers (Barker et al., 2009; Park et al., 2019). Interestingly, and consistent with our results, not all tumors that displayed elevated PheRS levels showed elevated levels of *α-PheRS* and *β-PheRS* mRNA. For instance, brain, ovary, endometrium and bladder tumors displayed only elevated *α-PheRS* mRNA levels, whereas colon, breast, lung and liver tumors showed elevated levels of mRNAs for both subunits. Because elevated levels of *α-PheRS* or *α-PheRS^Cys^* alone can elicit mitotic activity, growth and proliferation, our results suggest that the excessive PheRS (FARS) levels in tumor tissues might be able to produce such proliferative signals independent of whether they also produce elevated levels of β-PheRS. Modeling the effect of elevated α-PheRS levels in *Drosophila*, we found that elevated levels support growth and proliferation and lead to an increase in mitotic cells in different cell types. In follicle cells, more cells were produced in clones expressing more α-PheRS compared to wild-type clones. In wing discs, more mitotic cells were detected in most areas with higher levels of α-PheRS. This indicates that elevated α-PheRS levels can indeed be a risk factor for tumor formation in several different tissues.

## MATERIALS AND METHODS

### Fly genetics and husbandry

All *Drosophila melanogaster* fly stocks were kept for long-term storage at 18°C in glass or plastic vials on standard food with day/night (12 h/12 h) light cycles. All experiments were performed at 25°C with female animals unless specifically mentioned. A UAS-GFP element was added in experiments that tested for rescue and involved Gal4-mediated expression of the rescue gene. This construct served to even out the number of UAS sites in each Gal4-expressing cell. Origins of all stocks are provided in Table S2.

### DNA cloning and generation of transgenic flies

Sequence information was obtained from FlyBase. All mutations and the addition of the Myc tag to the N-terminus of *α-PheRS* were made by following the procedure of the QuickChange^®^ Site-Directed Mutagenesis Kit (Stratagene). The genomic *α-PheRS* rescue construct (*Myc::α-PheRS*) codes for the entire coding region and for an additional Myc tag at the N-terminal end. In addition, it contains ∼1 kb of up- and downstream sequences and it was cloned into the *pw*^+^*SNattB* transformation vector (Koch et al., 2009; Lu et al., 2014). The *α-PheRS* and *β-PheRS* cDNAs were obtained by RT-PCR from mRNA isolated from 4- to 8-day-old *OreR* flies (Lu et al., 2014). The Tyr412Cys and Phe438Cys mutations in the *α-PheRS* sequence were created by site-directed mutagenesis. Like the wild-type cDNA, they were cloned into the *pUASTattB* transformation vector to generate the pUAS-α-PheRS and pUAS-α-PheRS^Cys^. Before injecting these constructs into fly embryos, all plasmids were verified by sequencing (Microsynth AG, Switzerland). Table S4 lists the primers used to make the mutants and constructs, and the primers used to confirm their sequence. Transgenic flies were generated by applying the *φ* C31-based integration system with the stock (*y w att2A[vas-φ]; +; attP-86F*) (Bischof et al., 2007).

### Western blotting

Protein was extracted from tissues, whole larvae or flies using lysis buffer. Protein lysates were separated by SDS-PAGE and transferred onto PVDF membranes (Millipore, USA). Blocking was performed for 1 h at room temperature (RT) with non-fat dry milk (5%) in Tris-buffered saline with Tween (TBST) solution. Blots were probed first with primary antibodies (diluted in blocking buffer) overnight at 4°C and then with secondary antibodies (diluted in TBST) for 1 h at RT. The signal of the secondary antibody was detected using the detect solution mixture (1:1) (ECL™ Prime Western Blotting System, GE Healthcare Life Science) and a luminescent detector (Amersham Imager 600, GE Healthcare Life Science). Origins of reagents and recipes for buffers are provided in Tables S2 and S3, respectively.

### Immunofluorescent staining and confocal microscopy

Dissections were performed in 1× PBS on ice and tissue collected within a maximum of 1 h. Fixation was performed with 4% paraformaldehyde (PFA) in 0.2% phosphate buffered saline with Tween (PBST) for 30 min at RT (wing discs, ovaries). Then the samples were blocked overnight with blocking buffer at 4°C. Primary antibodies (diluted in blocking buffer) were incubated with the samples for 8 h at RT. The samples were rinsed three times and washed three times (20 min/wash) with PBST. Secondary antibodies (diluted in PBST) were incubated overnight at 4°C. The samples were then rinsed three times and washed two times (20 min/wash) with PBST. Hoechst 33258 (2.5 μg/ml) was added in PBST before the last washing step and the samples were mounted with Aqua/Poly Mount solution (Polysciences, USA). Origins and dilutions of all antibodies are provided in Table S2.

### Protein synthesis measurements using RPM

For puromycin labeling experiments, tissues were dissected in Schneider's insect medium (Sigma-Aldrich, USA) supplemented with 10% fetal calf serum (FCS; Sigma-Aldrich) at 25°C. They were then incubated with Schneider's insect medium containing puromycin (5 μg/ml, Sigma-Aldrich) and cycloheximine (100 µg/ml, Sigma-Aldrich) for 2 h at RT. Then the samples were fixed with 4% PFA in PBST 0.2% at RT and blocked overnight with blocking buffer at 4°C. Primary anti-Puromycin antibody (diluted in PBST) was incubated with the samples for 8 h at RT. The samples were rinsed three times and washed three times (20 min/wash) with PBST. Secondary antibodies (diluted in PBST) were incubated overnight at 4°C. The samples were then rinsed three times and washed two times (20 min/wash) with PBST. Hoechst 33258 (2.5 μg/ml) was added in PBST before the last washing step and the samples were mounted with Aqua/Poly Mount solution (Polysciences). See Table S2 for antibody details.

### *In vitro* aminoacylation assay

Recombinant α-PheRS and β-PheRS proteins were co-expressed in the *E. coli* strain Rosetta (Novagen) and then purified (Moor et al., 2002). For this, the *α-PheRS* or *α-PheRS^Cys^* mutant cDNAs were cloned with His tags at the N-terminal end into the pET-28a plasmid expression vector (Novagen). Wild-type *β-PheRS* cDNAs were cloned into the pET LIC (2A-T) plasmid (Addgene #29665). Then, His-α-PheRS or the His-α-PheRS^Cys^ mutant and β-PheRS were co-expressed in the *E. coli* strain Rosetta with isopropylthiogalactoside (1 mM) induction at 25°C for 6 h. Proteins were purified with Ni-NTA affinity resin (Qiagen). The aminoacylation assay protocol from Lu et al. (2014) was then followed, with the modification that the Whatman filter paper discs were soaked in phenylalanine solution for 1 h [30 mg/ml in 5% trichloroacetic acid (TCA)] to reduce the background. This assay was performed at 25°C in a 100-μl reaction mixture containing 50 mM Tris-HCl pH 7.5, 10 mM MgCl_2_, 4 mM ATP, 5 mM β-mercaptoethanol, 100 μg/ml BSA, 3 U/ml *E. coli* carrier tRNA, 5 μM [^3^H]-amino acid (L-Phe) and 1 μM tRNA^Phe^ from brewer's yeast (Sigma-Aldrich). In each experiment, a 15-μl aliquot was removed at four different incubation time points, spotted on the Phe-treated Whatman filter paper discs and washed three times with ice-cold 5% TCA and once with ice-cold ethanol. A blank paper disc without spotting and another with spotting of the enzyme-free reaction were used for detecting background signals. After filter discs were dried, they were immersed in PPO Toluol (Sigma-Aldrich) solution in plastic bottles and the radioactivity was measured by scintillation counting.

### Wing disc dissociation and fluorescence-activated cell sorting (FACS) analysis

Wandering larvae derived from 2-4 h egg collections were dissected in PBS during a maximal time of 30 min. Around 20 wing discs were incubated with gentle agitation at 29°C for ∼2 h in 500 µl 10× trypsin-EDTA supplemented with 50 µl 10× Hank's balanced salt solution (Sigma-Aldrich) and 10 µl Vybrant DyeCycle Ruby stain (Molecular Probes, USA). Dissociated cells from wing discs were directly analyzed by FACS-Calibur flow cytometer (Becton Dickinson, USA).

*Drosophila* tissue culture cells were harvested and fixed in 70% ethanol and stained with a staining solution containing 1 mg/ml propidium iodide, 0.1% Triton X-100 and 10 mg/ml RNase A. The cells were then subjected to FACS-Calibur cytometry and data were analyzed with the FlowJo software.

### *Drosophila* cell culture and RNAi treatment

*Drosophila* Kc cells were incubated at 25°C in Schneider's *Drosophila* medium supplemented with 10% heat-inactivated FCS and 50 µg/ml penicillin/streptomycin. To induce RNAi knockdown in *Drosophila* cells, dsRNA treatment was performed (Clemens et al., 2000). dsRNAs around 500 bp in length were generated with the RNAMaxx™ High Yield Transcription Kit (Agilent, USA). Cells were diluted to a concentration of 10^6^ cells/ml in serum-free medium, and dsRNA was added directly to the medium at a concentration of 15 µg/ml. The cells were incubated for 1 h followed by addition of medium containing FCS. Then the cells were kept in the incubator and were harvested at different time points 1-5 days after dsRNA treatment.

### Clonal assay and twin spot data analysis

For twin spot tests, we used the MARCM system. Twin spots were generated with the progenitor genotype *hs-flp; tub-Gal4/UAS-β-PheRS; FRT82B, ubiGFP, UAS-α-PheRS^(Cys)^/FRT82B Tub-Gal80.* In twin spots, the internal control clone was GFP-minus and the twin sister clone produced a red signal by the antibody against the overexpressed protein. We induced the *hs-FLP*, *FRT82B* system at 37°C for 1 h on the third day post-eclosure and dissected the animals 3 days post-induction. Confocal imaging detected non-green clones (without ubiGFP) and red clones (stained with anti-Myc antibody) (Fig. 5A).

In twin spots, cell numbers per clone were counted and the numbers of cell divisions per clone were calculated as log_2_(cell numbers per clone). This represents the logarithm of the cell numbers per clone to the base 2. The increase in cell proliferation (%) was analyzed by comparing the number of cell divisions of the clone pairs in the same twin spot. The clone sizes were measured by FIJI software, and the increase in clone size was analyzed by comparing the clone size in the same twin spot.

### Image acquisition and processing

Imaging was carried out with a Leica SP8 confocal laser scanning microscope equipped with a 405 nm diode laser, a 458, 476, 488, 496 and 514 nm Argon laser, a 561 nm diode-pumped solid-state laser and a 633 nm HeNe laser. Images were obtained with 20× dry and 63× oil-immersion objectives and 1024×1024 pixel format. Images were acquired using LAS X software. The images of the entire gut were obtained by imaging at the standard size and then merging maximal projections of *z*-stacks with the Tiles Scan tool. Fluorescence intensity was determined using FIJI software.

### Quantification and statistical analysis

For quantifications of all experiments, *n* represents the number of independent biological samples analyzed (the number of wing discs, the number of twin spots), error bars represent s.d. Statistical significance was determined using Student's *t*-test or ANOVA as noted in the figure legends.

## Supplementary Material

Supplementary information
