# Supplementary material for: A translation-independent function of PheRS activates growth and proliferation in Drosophila
Source: Dis Model Mech. 2021 Mar 18;14(3):dmm048132. doi: 10.1242/dmm.048132 (PMC7988764; doi:10.1242/dmm.048132)
Supplement: Supplementary information [file dmm-14-048132-s1.pdf]

Supplementary figures

Figure S1

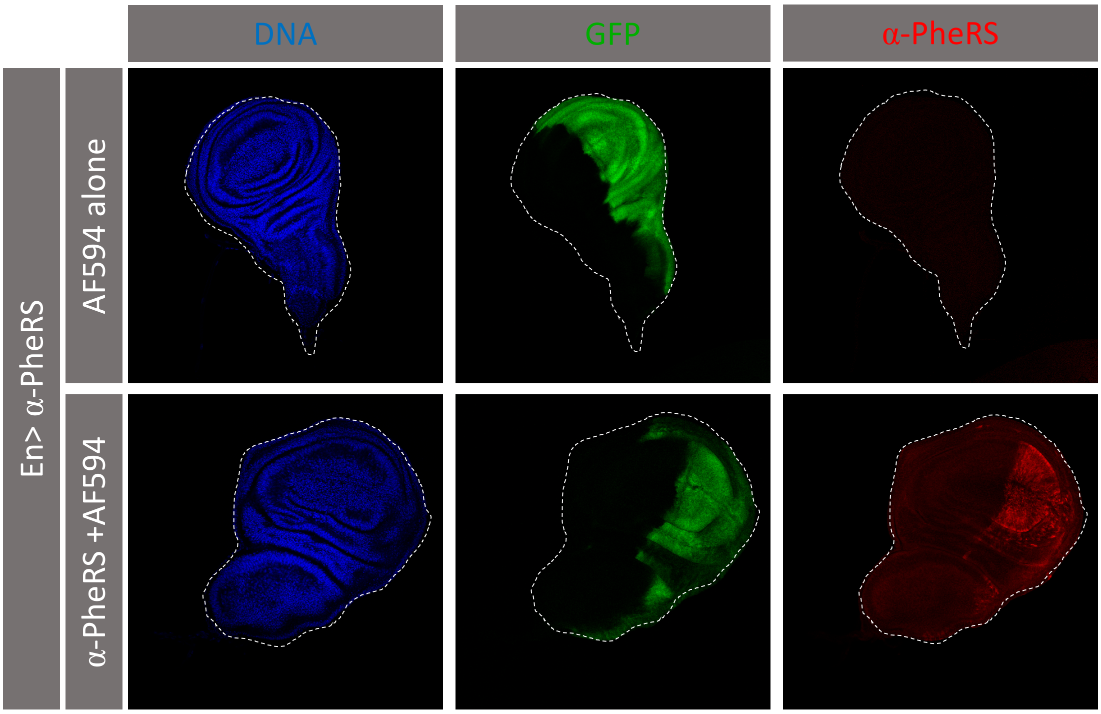

Figure S1: Measurement of additional expression of α-PheRS in the posterior wing disc compartment. The mean value of intensities of the fluorescent signals in the anterior and the posterior compartment were compared after applying the same settings (that did not produce a signal for the secondary antibody) for all image acquisitions. Pixel intensities in the two compartments of the wing discs were then compared. Driving the expression of only a wild-type copy of α-PheRS, the anti α-PheRS antibody produced a mean pixel intensity of 68 ± 9 in the posterior compartment and a mean pixel intensity of 37 ± 7 in the anterior compartment (n=4). The intensity was therefore 1,8 times as high in the posterior. The expression of a missense mutant of α-PheRS alone gave similar results. Posterior mean pixel intensity was 60 ± 13, the anterior one 34 ± 6; posterior levels were thus 1,7 times as high (n=8). Under the assumption that our primary antibody has 0%, 25% and 33%, respectively, cross reactivity, we measured and calculated an increase of the wild-type α-PheRS levels of 80%, 107%, and 120%, respectively. We have no evidence for high cross-reactivity of this antibody but cannot measure it because cells lacking α-PheRS are dead. For the mutant α-PheRS levels, the corresponding increase is 70%, 93%, and 105%, respectively.

Disease Models & Mechanisms • Supplementary information

Supplementary Figure S2

A) No or only minor negative correlation between protein expression levels and Phe frequency under high α-PheRS overexpression conditions

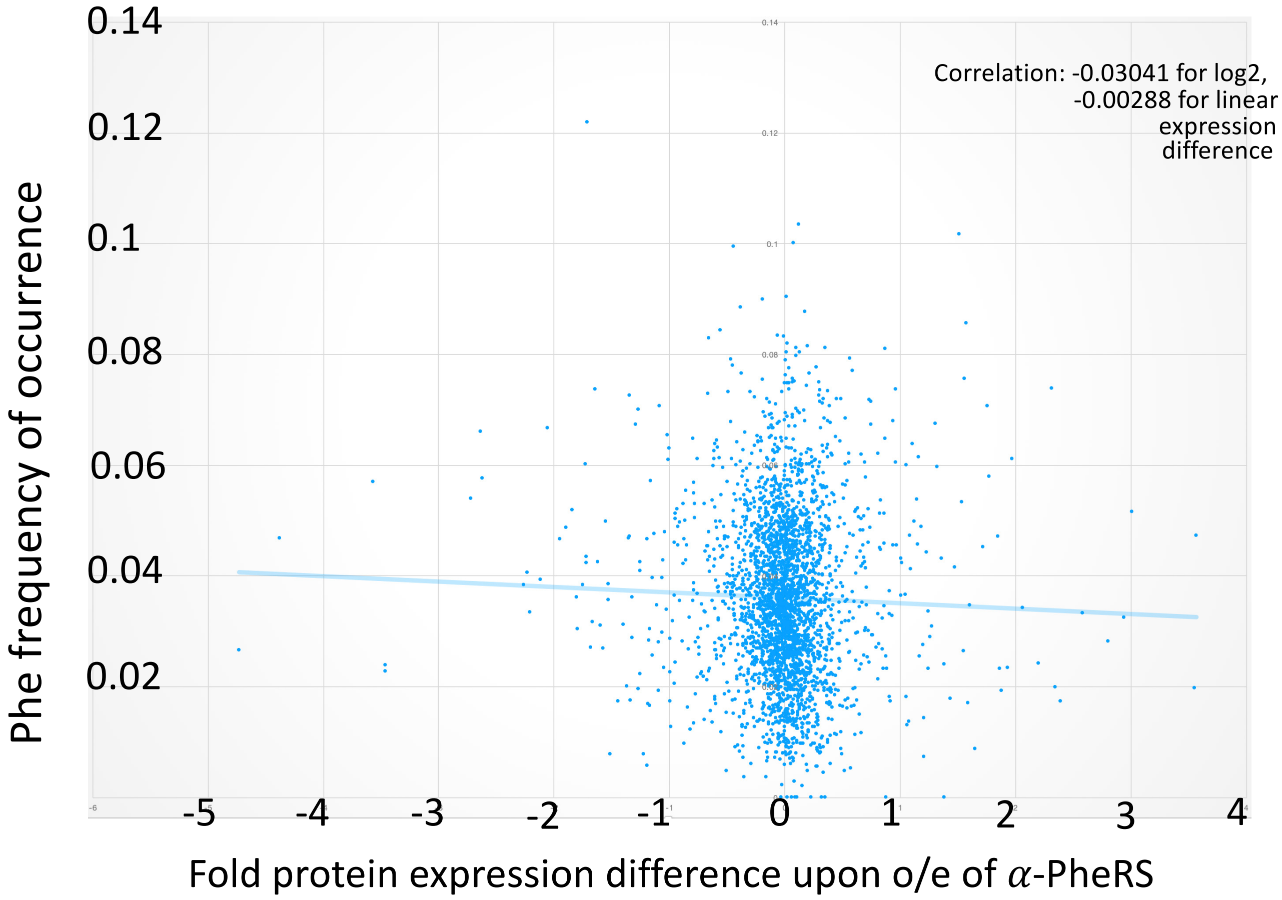

B) Phe codon frequency is only non-significantly higher and lower, respectively, in proteins that are less and more expressed, respectively, upon o/e of α-PheRS

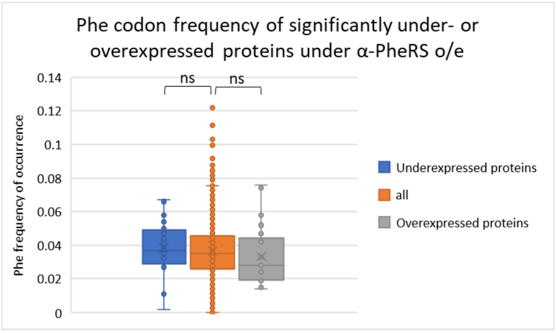

C) Overexpression of α-PheRS with the strong tub-Gal4 driver increases α-PheRS levels by 65% and reduces β-PheRS levels by 20%

Gene	Protein	Phes (#)	Length (aa)	Phenylalanine frequency	LFQ_eBayes_ log2-fold diff. α vs control	LFQ_eBayes adjpval α vs control
β-PheRS	Q9VCA5	26	589	0.044	-0.1979	0.31899
α-PheRS	Q9W3J5	24	498	0.048	0.6526	0.01268

Disease Models & Mechanisms • Supplementary information

Figure S2: No or only minor negative correlation between protein expression levels and Phe frequency under high α-PheRS over-expression conditions. Over-expression of one subunit of PheRS might affect the expression of the $(\alpha\beta)_2$PheRS complex that is required to load phenylalanine (Phe) onto the $tRNA^{Phe}$ producing the Phe-$tRNA^{Phe}$ needed for translation. Even though a general change in translation was not observed when translation was assessed in situ (Fig 3E), it is still conceivable that this might lead to a change in levels of Phe-$tRNA^{Phe}$ and uncharged $tRNA^{Phe}$ in the cell and that this might specifically affect translation of one or a few mRNAs with an extreme (probably more likely a high) frequency of Phe codons. To seek such hypothetical events that might be meaningful for growth and proliferation control, we systematically assessed the effect of overexpression of the α-PheRS subunit on the abundance of polypeptides with different and extreme frequencies of Phe codons using a proteomics approach. Towards this goal, the wild-type α-PheRS open reading frame was over-expressed under the strong α-tubulin84B-Gal4 >UAS control. First instar larvae were harvested, proteins were extracted and quantified using mass spectrometry. Experiments were carried out in biological triplicates and the identification of peptides and proteins was by Maxquant. The Maxquant Label-Free Quantification (LFQ) values were used to calculate the expression levels. The expression levels of these proteins were then compared to control larvae that expressed only the α-tubulin84B-Gal4 driver. Neither size nor p-value filters were applied to correlate this data with the Phe frequency of these proteins. To determine the Phe codon frequency, the UniProtKB 2020_05 database was used to determine the length and the number of Phe for each of the 22,045 Drosophila melanogaster proteins in the database. From this, the Phe frequency was established for each protein. **A)** The 2,986 proteins, for which LFQ enrichment could be determined, were used to analyze a possible correlation of enhancement of expression upon o/e of α-PheRS with relative abundance of Phe in the protein. This correlation analysis was performed in Excel. The result shows no or only a very weak negative correlation for this data set (-0.0029 for the linear expression difference and -0.0304 for the log2 fold expression difference). **B)** PheRS codon frequency does not change highly between the significantly under- or overexpressed proteins upon α-PheRS o/e. **C)** α-PheRS, with a log2 expression change of 0.652, does not show up in the significantly over-expressed proteins.

Disease Models & Mechanisms • Supplementary information

Table S1

Marked in red are proteins with a Phe frequency over the 75% quantile of all analyzed proteins and in blue the proteins with a Phe frequency under the 25% quantile of all analyzed proteins.

Psi has a role in tissue growth. It is also underexpressed in β-PheRS o/e. The effect is not specific for α-PheRS o/e

Significantly under-expressed upon alpha o/e			
**Leading Protein**	**gene**	**Known function (according to flybase)**	**Phe frequency value**
A1Z7F1	Mal-A6	Predicted to have maltose alpha-glucosidase activity. Predicted to be involved in carbohydrate metabolic process	0.038
A9J7N9	Aldh	Aldehyde dehydrogenase (Aldh) encodes an NAD[+] dependent mitochondrial aldehyde dehydrogenase. Its functions include detoxifying endogenous aldehydes generated by lipid peroxidation, and detoxifying acetaldehyde derived from dietary ethanol.	0.044
C5WLN1	Nadsyn	Predicted to have NAD+ synthase (glutamine-hydrolyzing) activity and glutaminase activity. Predicted to be involved in NAD biosynthetic process.	0.036
O18405	Surf4	unknown function, localizes to fusome	0.067
O76281	bt	A large protein associated with myosin thick filaments in insect muscles that contributes to the stiffness of flight muscle.	0.029
O96828	Psi	P-element somatic inhibitor (Psi) encodes a protein that has dual roles in RNA processing and transcriptional regulation. It is required for activating Myc transcription.	0.011
O97477	Inos	Exhibits inositol-3-phosphate synthase activity. Predicted to be involved in inositol biosynthetic process.	0.027
P04146	GIP	It is a transposable_element_gene from Dmel. It is encoded by the copia transposable element.. Its molecular function is described by: nucleic acid binding; zinc ion binding; aspartic-type endopeptidase activity. It is involved in the biological process described with: proteolysis; DNA integration.	0.05
Q7K1C3	CG6984	Predicted to have catalytic activity. Predicted to localize to mitochondrion.	0.032
Q7KR10	Cyp12d1-d	Cyp12d1-d (Cyp12d1-d) encodes a protein involved in the response to the insecticide dichlorodiphenyltrichloroethane (DDT)	0.058
Q86BM0	CG9515	Predicted to have nucleoside-triphosphate diphosphatase activity.	0.038
Q86PC7	anon1A3	unknown function	0.002
Q8ML70	IMPPP	Involved in defense response; humoral immune response; and response to bacterium.	0.047
Q8T3T8	SD06908p	unknown function	0.029
Q966T5	Pax	Paxillin (Pax) encodes an adaptor protein that is generally associated with integrins. It is not required for most integrin-mediated adhesion or migration events, but is required for viability beyond pupal stages. It controls the size of some muscles by regulating the rate of cell fusion.	0.066
Q9NHF8	gag	unknown function	NA
Q9VLL5	ESTS:172F5T	Predicted to have alpha-N-acetylglucosaminidase activity	0.054
Q9VMZ6	TwdlG	Predicted to be a structural constituent of chitin-based cuticle. Predicted to be involved in body morphogenesis and chitin-based cuticle development.	0.029
Q9VNR7	CG11241	Predicted to have alanine-glyoxylate transaminase activity.	0.033
Q9W095	Gk2	Exhibits glycerol kinase activity. Involved in glycerol-3-phosphate biosynthetic process and phosphorylation.	0.035
Q9W3K9	Ldsdh1	Predicted to have retinol dehydrogenase activity.	0.041

Disease Models & Mechanisms • Supplementary information

Significantly over-expressed upon alpha o/e			
Leading Protein	gene	Known function (according to flybase)	Phe frequency value
A0A1B2AKC6	ND-ACP	Predicted to have acyl binding activity; acyl carrier activity; and phosphopantetheine binding activity. Predicted to be involved in fatty acid biosynthetic process and mitochondrial electron transport, NADH to ubiquinone. Predicted to localize to mitochondrial respiratory chain complex I.	0.042
Q7K1U0	Arc1	Exhibits mRNA binding activity. Involved in several processes, including behavioral response to starvation; mRNA transport; and vesicle-mediated intercellular transport.	0.047
Q7JX43	Jonah 44E	Predicted to have serine-type endopeptidase activity. Predicted to be involved in proteolysis.	0.033
Q7K1Q6	Jabba	Jabba (Jabba) encodes a lipid droplet protein that sequesters histones on lipid droplets. It promotes proper development of pre-cellular embryos, controls levels of nuclear histones, and mediates anti-bacterial protection.	0.028
C7LAA5	CG30122-RB	Predicted to be involved in mRNA splicing, via spliceosome. Localizes to precatalytic spliceosome.	0.017
Q9VXY7	Lsd-2	Lipid storage droplet-2 (Lsd-2) encodes a protein associated with lipid droplets. It acts as a barrier for lipases (such as the product of bmm) and thus prevents the mobilization of lipid stores. It is involved in regulation of lipid storage amount and energy homeostasis and acts in concert with the product of Lsd-1.	0.017
Q9W3L4	CG2233	unknown function	0.058
E1UI91	CG10912	Involved in cold acclimation.	0.03
Q8SZN1	CG31313	Predicted to have cysteine-type endopeptidase inhibitor activity.	0.024
Q7K3N4	CG8888	Predicted to have estradiol 17-beta-dehydrogenase activity and testosterone dehydrogenase (NAD+) activity.	0.052
A0A0B4LF52	betaTry	βTrypsin (βTry) encodes a putative digestive enzyme with predicted serine-type endopeptidase activity. βTry is highly expressed and enriched in the midgut, an organ involved in digestion.	0.02
Q9VY04	fiz	Predicted to have ecdysone oxidase activity. Involved in mesoderm development.	0.033
A8E6I8	thetaTry	θTrypsin (θTry) encodes a putative digestive enzyme with predicted serine-type endopeptidase activity. θTry is highly expressed and enriched in the midgut, an organ involved in digestion.	0.015
M9PDP6	CT31762	Predicted to have calcium ion binding activity.	0.019
Q9VMT6	Cyp28d2	Predicted to have heme binding activity; iron ion binding activity; and oxidoreductase activity. Predicted to be involved in oxidation-reduction process	0.074
Q9VYU9	CG9360	Predicted to have 17-beta-hydroxysteroid dehydrogenase (NADP+) activity; 17-beta-ketosteroid reductase activity; and 3-keto sterol reductase activity	0.02
M9MS77	CG42704	unknown function	0.076
Q9VMX8	Jon25Bii	Predicted to have serine-type endopeptidase activity. Predicted to be involved in proteolysis	0.025
Q9VUX0	hzg	Predicted to have RNA polymerase II CTD heptapeptide repeat phosphatase activity. Predicted to be involved in protein dephosphorylation.	0.03
E8NH67	aspr	Its molecular function is unknown. It is involved in the biological process described with: positive regulation of Notch signaling pathway; tissue regeneration	0.014
O16101	Jon25Bi	Predicted to have serine-type endopeptidase activity. Predicted to be involved in proteolysis.	0.026

Table S2. Resources and reagents

Reagent or resource	Source	Identifier	Additional information
Antibodies			
Anti phospho-Histone H3-rabbit	Cell signaling	9701S	1:200 v/v
Anti phospho-Histone H3-mouse	Cell signaling	9706S	1:200 v/v
Anti *α-PheRS*	Genescript	4668	Customized product (1:200 v/v)
Anti *α-PheRS*	Genescript	4669	Customized product (1:200 v/v)
Anti Myc-mouse	Developmental Studies Hybridoma Bank (DSHB)	9E10	Supernatant (1:3 v/v)
Anti Puromycin	DSHB	PMY-2A4	1:100 v/v
Anti Cy3 rabbit	Jackson Immuno Research	115-165-146	1:200 v/v
Anti-rabbit Alexa Flour 488	Molecular Probes	A-11008	1:200 v/v
Anti-rabbit Alexa Flour 488	Molecular Probes	A-11034	1:200 v/v
Anti-mouse Alexa Flour 488	Molecular Probes	A-11029	1:200 v/v
Anti-rabbit Alexa Flour 488	Life technology	A-21206	1:200 v/v
Anti-rabbit Alexa Flour 594	Invitrogen	A-11037	1:200 v/v
Anti-mouse Alexa Flour 594	Molecular Probes	A-11032	1:200 v/v
Anti-mouse Alexa Flour 568	Life technology	A-10037	1:200 v/v
Anti actin	Abcam	Ab18251	1:1000 v/v
Anti GFP	ImmunoKontact	042704	1:1000 v/v
Anti Myc-rabbit	Santa Cruz	Sc-789	A-12 (1:1000 v/v)
HRP Anti rabbit IgG antibody (Peroxidase)	Vector	PI-1000	1:10,000 v/v
HRP Anti rabbit IgG antibody (Peroxidase)	Vector	PI-2000	1:10,000 v/v
Fly stocks and genetics			
α-PheRS^{G2060}*/FM6*	Bloomington Drosophila Stock Center (BDSC)	26625	
RNAi- α-PheRS	Vienna Drosophila RNAi Center (VDRC)	33514	
RNAi- β-PheRS	VDRC	42046	
gα-PheRS^{Cys}			Transgenic construct
UAS-α-PheRS^{Cys}			Transgenic construct
hspFLP; Act-Gal4/CyO; neoFRT82B,tub-Gal80/TM3,Sb			
w; If/CyO; neoFRT82B,UAS- α-PheRS$^{(Cys)}$			
eyeless-Gal4	BDSC	5535	
ppl-Gal4	BDSC	58768	
engrailed-Gal4	BDSC	30564	
UAS-GFP	BDSC	6658	
w; UAS-Myc::MYC	BDSC	9674	
hspFLP/y; +; UAS-Myc::MYC	BDSC	9675	
neoFRT82B Sb1/TM6	BDSC	2051	
tub-Gal4/TM3,Sb	BDSC	5138	
y w att2A[vas-ϕ]; +; attP-86F	ETH Zurich		A gift from Hugo Stocker, ETH
yw;UAS-cyto-gars-myc/CyO			A gift from Albena Jordanova, VIB-U Antwerp Center for Molecular Neurology
Bacteria strains and vectors			

Reagent or resource	Source	Identifier	Additional information
XL1 blue	Agilent	200249	
Rosseta – Novagen	Merckmilipore	70954	
pET-28a – Novagen	Merckmilipore	69864	
pET LIC (2A-T)	Addgene	29665	
pUASattB	Drosophila Genomics Resource Center	1419	
pw+SNattB	(Koch et al., 2009)		
Commercial assay or kit			
Pierce® Silver Stain kit	Thermo Scientific	24612	
Pierce® BCA Protein Assay kit	Thermo Scientific	23227	
ReliaPrep™ DNA CleanUp and Concentration System	Promega	A2893	
GeneElute™ HP Plasmid miniprep kit	Sigma	NA0160	
Qiagen® Plasmid Plus Midi kit	Qiagen	12943	
Ni-NTA affinity resin	Qiagen	30210	
ECL™ Prime Western Blotting System	GE Healthcare	RPN2232	
RNAMaxx™ High Yield Transcription Kit	Agilent	200339	
Software, algorithm			
Leica Application Suite X (LAS X)	Leica	https://www.leica-microsystems.com/products/microscope-software/p/leica-las-x-ls/	
FIJI	ImageJ	https://fiji.sc/	
GraphPad Prism	GraphPad	https://www.graphpad.com/scientific-software/prism/	
FlowJo™	BD Biosciences	https://www.flowjo.com/	
Microsoft Excel	Microsoft	https://products.office.com/en-us/excel	

Disease Models & Mechanisms • Supplementary information

Table S3. Buffers

Buffers	
Lysis buffer for Drosophila tissue	Lysis buffer for bacteria
20 mM Tris HCl pH7.4<br>150 µM NaCl<br>2 mM EDTA<br>50 mM NaF<br>10% Glycerol<br>1% Triton X100<br>1 Protease inhibitor cocktail tablet<br>(Roche-4693159001)<br>1 mM phenylmethylsulphonyl fluoride	20 mM Tris HCl pH7.4<br>150 µM NaCl<br>2 mM EDTA<br>50 mM NaF<br>10% Glycerol<br>1% Triton X100<br>4 mM Imidazole 1M<br>0.6% Lysozyme<br>1 Protease inhibitor cocktail tablet<br>1 mM phenylmethylsulphonyl fluoride
4% PFA	1× PBST
1× PBST<br>4% (w/v) Paraformaldehyde	0.2% (v/v) Tween 20<br>1× PBS
Blocking buffer	Fly food recipe
5% (w/v) non-fat dry milk<br>0.1% (v/v) Triton X100	20.4 l H_2O<br>1680 g Maize flour<br>720 g Yeast<br>1800 g Syrup<br>192 g Potassium sodium tartrate tetrahydrate<br>36 g Nipagin<br>120 ml Propionic acid
10× PBS pH 7.4	
10.6 mM KH_2PO_4<br>1.5 M NaCl<br>30 mM $Na_2PO_4.7H_2O$	
10× SDS running buffer	10× Transfer buffer
30 g Tris base<br>144 g Glycine<br>10 g SDS<br>dH_2O to 1 l	30 g Tris base<br>144 g Glycine<br>dH_2O to 1 l
10× TBS pH to 7.6	1× TBST
24 g of Tris Base<br>88 g of NaCl<br>dH_2O to 1 l	100 ml 10× TBS<br>900 ml dH_2O<br>0.1% (v/v) Tween 20

Table S4. Primers

Primers		
Name	Sequence (5′ to 3′)	Application
rc2263f	CGCGGATCCATCCGGCGAGAGAGTGTCTTTG	Genomic genomic construct of *α-PheRS*
rc2263r	CGGGGTACCTATGCCTGGCGATAATCGTG	
Tyr412Cys & Phe438Cys-F	TCAAGCCGGCGTACAATCCGTGTACCGAGCCCAG	Construct of *α-PheRS^{Cys}* mutation
Tyr412Cys & Phe438Cys-R	CTCCGGCCGACAGACGCCCGAGTTGCCC	
α-PheRS RNAi 11f	TAATACGACTCACTATAGGGAGGCAAGAAACGCAAGCTCCTC	*α-PheRS* ds RNA synthesis
α-PheRS RNAi 11r	TAATACGACTCACTATAGGGAGGGAACTCCGCCAGATGTGTG	
β-PheRS RNAi 10f	TAATACGACTCACTATAGGGAGGGCCAATCATTCGGGAATCA	*β-PheRS* ds RNA synthesis
β-PheRS RNAi 10r	TAATACGACTCACTATAGGGAGGAGGCAGGGACTTCTTAATGT	
seq r6	GCTCCCATTCATCAGTTCC	Sequencing
seqA r1	CATTTCCACCGTGAGATCCGTC	Sequencing
seqA r2	AACTCTTGTGGGTGACCGTTTC	Sequencing
seqA f1	GTTCTCGAAGTGAATGTTCTGG	Sequencing
seqA f2	TTTAGCCACCGTCGTCGTTTC	Sequencing
seqA r3	TCCAGCGACGATGACGAATTTG	Sequencing
seqA f3	CAAATGGATTGTGGGACCAGC	Sequencing
seqA r4	GCCCTCCTCCACCATCTTTAG	Sequencing

Disease Models & Mechanisms • Supplementary information
